# Bayesian analysis of high-resolution ultrasonography and guided fine needle aspiration cytology in diagnosis of palpable thyroid nodules^☆^

**DOI:** 10.1016/j.bjorl.2016.10.010

**Published:** 2016-11-17

**Authors:** Niranjan Sahu, Rabindra Nath Padhy

**Affiliations:** a Siksha ‘O’ Anusandhan University, Institute of Medical Sciences and Sum Hospital, Department of Radiodiagnosis, Odisha, India; b Siksha ‘O’ Anusandhan University, Institute of Medical Sciences and Sum Hospital, Central Research Laboratory, Odisha, India

**Keywords:** Malignant thyroid nodule, Colloid goiter, High-resolution ultrasonography, Bayes rule, Sensitivity and specificity, Nódulo maligno da tireoide, Bócio coloide, Ultrassonografia de alta resolução, Regra de Bayes, Sensibilidade e especificidade

## Abstract

**Introduction:**

To evaluate diagnostic accuracy of high-resolution ultrasonography in differentiation of benign and malignant thyroid nodules in comparison to results of guided fine needle aspiration cytology based on the Bayes rule.

**Objective:**

To assess the validity of ultrasonography results of thyroid nodules in comparison to guided fine needle aspiration cytology findings.

**Methods:**

This study was done on randomly chosen 80 patients presented with palpable thyroid nodules, undergone real-time sonographic evaluation of thyroid nodules to characterize features, internal consistency, margins, echotexture, calcification, peripheral lucent halo and vascularity. Ultrasonography guided fine needle aspiration cytology studies of thyroid nodules were done.

**Results:**

Palpable thyroid nodules were highly prevalent in fourth and fifth decades of life with female–male ratio, 4:1. Solid internal consistency was demonstrated by 75% malignant nodules. Hypoechogenicity and intra-nodular micro-calcifications were observed in 92% malignant nodules; 83% malignant nodules had intra-nodular vascularity and absence of peripheral halo. The pre-test prevalence of malignant nodules in the targeted population was 17.5%. As type I error, 2.5% false-positive cases and as type II error, 5.0% false-negative cases were detected. Values of sensitivity and specificity of the ultrasonography test were 71.43 and 96.97%, respectively.

**Conclusion:**

Malignant thyroid nodules demonstrated ultrasonography characteristics of hypoechoic texture, intra-nodular micro-calcifications, solid consistency, internal vascularity and absence of peripheral halo. The ultrasonography test has 92.5% diagnostic accuracy to differentiate malignant from benign lesions in comparison to the gold standard fine needle aspiration cytology test.

## Resumo

**Introdução:**

Avaliar a precisão diagnóstica da ultrassonografia de alta resolução na diferenciação de nódulos tireoidianos benignos e malignos em comparação com os resultados da citologia de aspiração por agulha fina baseada na regra de Bayes.

**Objetivo:**

Avaliar a validade dos resultados da USG de nódulos da tireoide em comparação com os resultados obtidos por citologia de aspiração por agulha fina.

**Método:**

Esse estudo foi realizado em 80 pacientes selecionados aleatoriamente, que apresentavam nódulos palpáveis da tireoide, submetidos à avaliação ultrassonográfica em tempo real de nódulos da tireoide para estabelecer características, consistência interna, margens, ecotextura, calcificação, halo lucente periférico e vascularização. Foram realizados estudos por citologia de aspiração por agulha fina guiados pela USG dos nódulos de tireoide.

**Resultados:**

Nódulos palpáveis da tireoide foram altamente prevalentes na quarta e quinta décadas de vida com uma razão sexo feminino-masculino de 4: 1. A consistência interna sólida foi demonstrada em 75% de nódulos malignos. Hipoecogenicidade e microcalcificações intra-nodulares foram observadas em 92% de nódulos malignos; 83% dos nódulos malignos apresentaram vascularidade intra-nodular e ausência de halo periférico. A prevalência pré-teste de nódulos malignos na população alvo foi de 17,5%. Como erro tipo I, houve 2,5% de casos falso-positivos e como erro tipo II, foram detectados 5,0% de casos falso-negativos. Os valores de sensibilidade e especificidade do exame por USG foram de 71,43 e 96,97%, respectivamente.

**Conclusão:**

Nódulos malignos da tireoide mostraram características de textura hipoecoica, microcalcificações intra-nodulares, consistência sólida, vascularidade interna e ausência de halo periférico na USG. O exame por USG tem 92,5% de precisão diagnóstica para diferenciar lesões malignas de benignas em comparação com o padrão ouro da citologia de aspiração por agulha fina.

## Introduction

Thyroid masses are usually abnormal glandular swellings, comprising of either nodular or diffuse enlargements of the gland. Diffuse thyroid masses are usually due to benign causes such as, hyperplastic colloid goiter, chronic Hashimoto's thyroiditis and Grave's disease.^1^ Nodular thyroid masses (TNs) show 64.6% prevalence in general population at autopsy.^2^ As the majority of palpable thyroid nodules are benign in nature with relative rarity of malignancy, a reliable method for differentiating clinically significant malignant nodules from innocuous benign ones is desirable. Early diagnosis of malignant thyroid nodules is coveted, because of good post-surgical prognosis.

The high-resolution real-time gray-scale ultasonography (USG) method has simplified the evaluation of the anatomy of normal thyroid and pathologic conditions, with remarkable clarity; consequently, an accurate assessment of the associated lesions can be done, because of superficial location of the thyroid gland. Detection of TNs by USG had been reported to have increased from 19–68% with the technological development of ultrasound equipments.^2^ USG being a quick and non-invasive procedure is the most accepted imaging modality nowadays, and as a non-ionizing technique it remains safe for children and pregnant women. Other patient-friendly advantages are its ready availability, low cost and non-requirement of any clinical manipulation with the patient prior to carrying out the procedure. Moreover, Thyroid Imaging Reporting and Data System (TI-RADS) is a USG based study known for a better stratification of sonographically suspicious features of malignancy. Furthermore, the USG guided technique for FNAC procedure is concomitantly helpful in obtaining adequate specimens even from small thyroid nodules. Today, high-frequency transducers (10–13 MHz) provide a greater degree of spatial resolution. On the other hand, medium-frequency transducers (5–6.5 MHz) present a good compromise between resolution as well as penetration of deeper structures of neck.

In this report, an evaluation of the diagnostic accuracy of high-resolution USG findings in the differentiation of benign and malignant thyroid nodules, as well as the sensitivity and specificity of USG in comparison to guided FNAC results, are presented. The accuracy of a patient-friendly diagnostic test, USG can be measured by comparing with the results of the available gold standard test, FNAC. From the data the former appears more undependable than the FNAC test. The available data were analyzed using the Bayes rule to assess the USG test, as a preferential diagnostic imaging modality. It is anticipated that this lamplit analysis would be helpful in quantifying pervasive errors in these conventional diagnostic methods for thyroid malignancy. Consequently, time and resources for treating false-positives (FP) or ‘type I error’ (USG test malignancy, FNAC test benign) cases with dogged cancer therapy would not be wasted, and the spate of non-target complications of the treatment could be blithely avoided. Moreover, false-negative (FN) or ‘type II error’ (USG test benign, FNAC test malignancy) cases should not be neglected, since a fear of future malignancy is generated. However, true-positives (TP) (USG test malignancy, FNAC test malignancy) and true-negatives (TN) (USG benign, FNAC test benign) are the sought-after results of a correct diagnosis. This study further digitally compares USG findings with a bond wagon of test statistics with the gold standard (fine needle aspiration cytology) FNAC test confirming the use of USG in the phase of basic problem of interface of FNAC when rarely warranted. Thus, a chiaroscuro-like analysis of these popular diagnostic test methods for palpable thyroid masses using the Bayes rule is essential for assessing the diagnostic accuracy of these tests; but that being unavailable in literature, this work is anticipated to strengthen confidence levels on these tests in deciding whether an invasive corrective measure would be need for a thyroid mass.

### What Bayesian analysis can do?

Diagnostic tests are used for revealing the occurrence of randomly distributed diseased individuals having both malignant and benign (absence of malignancy) thyroid nodules. The accuracy of a diagnostic test can be measured by comparing the test results with the true condition of patients individually. Herein, the ambivalence of USG and FNAC test results for comparative accuracy could be resolved with the account of data as evidence, by an appropriate statistical analysis involving probability – as how much each test is dependable. A clinician would be always eager to know numerically about the errors of each test, at one's laboratory condition. Obviously, the FNAC test is assumed as the gold standard, since it reveals the true condition of a patient. Thus, it is an ideally based truth with which, the second test, being user-friendly and patient-friendly can be compared for digital values as a method. Thus, the USG test is the candidate to be assessed for its sufficiency as a diagnostic test. However, the FNAC test also has a degree of unreliability, not having representable or adequate thyroid tissue from the appropriate nodule consequent to improper placement of guided needle tip due to technical error. Thus prudently, the Bayesian analysis based on obtained data as evidence could measure the degree of belief/assumption, for what percent the FNAC test could be taken as gold standard, and concomitantly, how much numerically the USG test would be dependable. To evaluate the inherent probability of each, the prior probability (a priori probability or prevalence or the prevalence of thyroid malignancy in the targeted population) was determined before using data, prevalence = [(TP + FN)/*n*], with *n* = number of total number of thyroid masses. And both tests are independent by themselves, but are critical in determining the status of each test. Furthermore, there are several associated test statistics: the sensitivity (true positive rate) – this is the portion of the people with the disease (malignancy), who will have positive USG test results, computed by [TP/(TP + FN)], and the specificity (true negative rate) – this is the portion of the people without the disease, who will have benign nodule in USG test results, computed by [TN/(FP + TN)]; these test statistics are bases of the Bayesian analysis.^3^

Furthermore, the false positive rate – it is the probability of errors of the FNAC test, computed by [FP/(FP + TN)], and the false negative rate – it is the probability of errors of the USG test, computed by FN/(TP + FN) are important. And the positive predictivity – it is the post-test probability of the disease that gave a positive test result or this is the portion of the people who actually have thyroid malignancy, computed by [TP/(TP + FP)], predicted positivity by the USG test; and the negative predictivity – post-test probability of the disease that gave a negative test result or this is the portion of the people negative for malignancy, computed by [TN/(FN + TN)], predicted negativity (absence of malignancy) by the USG test. Also the diagnostic accuracy (inherent validity or predictive validity) – it is the ability of the USG test to be correctly positive or negative, computed by [(TP + TN)/*n*]. Furthermore, the positive likelihood ratio is the ratio between TP rate and FP rate, computed by [sensitivity/(1 − specificity)], when the USG test result is positive; and the negative likelihood ratio is the ratio between FN rate and TN rate, computed by [(1 − sensitivity)/specificity], when the USG test result is benign. The larger the positive likelihood ratio value, the greater is the likelihood of malignant nodules, and similarly, the smaller the negative likelihood ratio value, the lesser is the likelihood of malignancy, in a population. And a posteriori probability is the value from post-test arithmetic computation of the data for diagnostic efficiency, and it specifically analyses how much (numerically) good/dependable the test is, independently in arriving at the truth–the coveted conclusions from these tests on individual patients.

## Methods

The present work on high-resolution real-time sonographic evaluation of palpable thyroid nodules was carried out in Department of Radiodiagnosis. It is a prospective study conducted on randomly chosen 80 patients of both sexes presented with palpable thyroid nodules, over a 2 year period (Feb 2013–Dec 2014) in the eastern costal region of India. The correlation of USG findings with guided FNAC results were carried out. The study excluded cases having questionably palpable thyroid nodules, diffuse thyroid masses and chronic renal failure, in which parathyroid nodules may coexist.

Real-time sonographic evaluation was performed on MEDISON ACCUVIX A30 Scanner, installed in the department using a linear transducer having wide range frequency of 5–13 MHz providing both high resolution and deeper penetration as well. Evaluation of thyroid nodules were done to characterize sonographic features such as, internal consistency (solid/mixed cystic/cystic), margins (regular/irregular), echotexture (hyper/iso/hypo/hetero/anechoic), calcification (coarse/fine), peripheral lucent halo (thick/thin, complete/incomplete) and intralesional vascularity status on Color Doppler Flow Imaging (CDFI). The presence of additional features, intra-nodular hemorrhage, comet-tail artifacts and cervical lymphadenopathy were also monitored. USG findings were correlated with guided FNAC reports to determine its sensitivity and specificity values.

## Results

Usually TNs measuring above 1.5 cm are palpable clinically. The age of the total 80 patients presented with palpable TNs ranged from 21–60 years, with a female–male ratio of 4:1. In the age group of 31–40 years, 38 (47.5%) cases and in the age group of 41–50 years, 28 (35%) cases of the total 80 cases had palpable TNs (Table 1). Moreover, at 21–50 year group, 95% benign cases were noted. Furthermore, 92% cases of malignant nodules were presented in the fifth and sixth decades of life with a female male ratio of 3:1.

**Table 1:** Age and sex distribution of thyroid nodules of 80 cases.

Sex	21–30 y	31–40 y	41–50 y	51–60 y	Total
Male	02	07	06	01	16 (20)
Female	08	31	22	03	64 (80)
Total	10 (12.5)	38 (47.5)	28 (35)	04 (5)	80 (100)

y, years; numbers in parenthesis are percent values.

Of the total 80 cases, a major fraction of 68 (85%) cases were benign, of which only hyperplastic colloid nodules comprised of 64 cases. Malignant nodules were detected in 12 (15%) cases. Only 71% of the total thyroid nodules were solid, while 24% nodules exhibited mixed cystic changes. Nine out of 12 cases among malignant nodules demonstrated solid consistency. Hypoechogenicity was observed in 11 out of 12 malignant nodules; and half of the total benign nodules, demonstrated hyperechogenicity. Incidences of intra-nodular calcification were recorded in 92% malignant nodules and 31% benign nodules. Furthermore, thick incomplete or absence of perinodular sonolucent halo was noted in 10 out of 12 malignant cases. Intra-nodular vascularity was too observed in 10 out of 12 malignant nodules, while the rest 2 cases had peripheral vascularity. Cervical lymphadenopathy was demonstrated by 10 malignant cases out of which, only 2 cases had cystic consistency (Table 2).

**Table 2:** Number of patients with different ultrasonography (USG) features of benign and malignant nodules, distributed among 80 cases.

USG features	Benign nodules	Malignant nodules
*Internal consistency*		
Solid	48	09
Mixed cystic	16	03
Cystic	04	–

*Margins*		
Regular	50	02
Irregular/microlobulated	04	10

*Echotexture*		
Hyperechoic	32	–
Isoechoic	18	–
Hypoechoic	02	11
Heteroechoic	12	01
Anechoic	04	–

*Calcification*		
Coarse (macro)	20	–
Fine (micro)	01	11

*Peripheral lucent halo*		
Thin complete	56	01
Thick incomplete/absence	02	10

*Vascularity*		
Peripheral	36	02
Internal	08	10
Avascular	24	–
Lymphadenopathy	06	08

–, not found.

Of the total of 80 (1.0) evaluated cases, it was found that 12 (0.15) cases were with malignant nodules and the rest 68 (0.85) cases had benign nodules in USG test results; whereas 14 (0.175) cases were malignant from FNAC test results, and the rest 66 (0.825) cases had FNAC-benign nodules; test results of all cases got Balkanized to four categories as TP, TN, FP and FN cases in Table 3. In other words, there was a mismatching of USG test and FNAC test results. The total of 14 malignant (TP + FN) cases signify that the prevalence of malignancy was 17.5% (probability value, 0.175), in the targeted population of 80 cases.

**Table 3:** The generic 2 × 2 table with number of cases assigned to malignant and benign nodules, based on USG test and fine needle aspiration cytology (FNAC) test during diagnosis of thyroid nodules.

USG test results	FNAC test results		Total
	Malignant nodules	Benign nodules	
Malignant nodules	TP = 10 (0.125)	FP = 02 (0.025)	(TP + FP) = 12 (0.15)
Benign nodules	FN = 04 (0.05)	TN = 64 (0.8)	(FN + TN) = 68 (0.85)
Total	(TP + FN) = 14(0.175)	(FP + TN) = 66 (0.825)	*n* = 80 (1.0)

TP, 10 cases were true-positives (USG-malignancy, FNAC-malignancy); FP, 02 cases were false-positives (USG-malignancy, FNAC-benign); FN, 04 cases were false-negatives (USG-benign, FNAC-malignancy); and TN, 64 samples were true-negatives (USG-benign, FNAC-benign); *n*, population size or total number of cases. Corresponding fraction values are given in parentheses. Prevalence of thyroid malignancy in the population was 0.175. FP cases are the type I errors, while FN cases are the type II errors.

The sensitivity value is the ability of the USG test to detect the malignancy status, when it is truly present, i.e., it is the probability of a ‘positive test result’. On the other hand, the specificity value is the ability of the USG test to give the negative result with malignancy-free individuals, i.e., it is the probability of a ‘negative test result’. Additionally, the diagnostic accuracy value estimates accuracy of both USG and FNAC tests together. And applying the Bayesian concept with these recorded data (Table 3), a bandwagon of other test statistics could be computed for additional probability values, along with their corresponding 95% confidence interval (CI) values (Table 4).

**Table 4:** Computed probability values of different Bayesian test statistics of diagnosis of thyroid tumors.

Test statistic	Formula	Value	95% CI
Prevalence or a priori probability	(TP + FN)/N	0.1750	0.7560–1.1390
Sensitivity (true positive rate)	TP/(TP + FN)	0.7143	0.8388–1.0612
Specificity (true negative rate)	TN/(FP + TN)	0.9697	0.9139–1.9860
Diagnostic accuracy	(TP + TN)/N^a^	0.9250	0.8930–1.0010
Positive predictivity	TP/(TP + FP)	0.8333	0.8651–1.0349
Negative predictivity	TN/(FN + TN)	0.9412	0.8996–1.0040
False positive rate	FP/(FP + TN) = (1 − specificity)	0.0303	0.7451–1.1549
False negative rate	FN/(TP + FN) = (1 − sensitivity)	0.2857	0.7741–1.1259
Positive likelihood ratio	Sensitivity/(1 − specificity)	2.3573	0.7090–1.1910
Negative likelihood ratio	(1 − sensitivity)/specificity	0.2946	0.7752–1.1248
A posteriori probability	*p*(*E*_1_|*E*)	0.5647	0.8127–1.0873

CI, confidence interval. For the detailed formula of a posteriori probability see text.

a Alternately, (sensitivity) (prevalence) + (specificity) (1 − prevalence).

### A posteriori probability

The a posteriori probability or ‘*p*(*E*_1_|*E*)’, the probability value of a thyroid mass to be truly positive, could be calculated by the Bayesian formula, *p*(*E*_1_|*E*) = *p*(*E*_1_) × *p*(*E*|*E*_1_)/[*p*(*E*_1_) × *p*(*E*|*E*_1_) + *p*(*E*′_1_) × *p*(*E*|*E*′_1_)], where, *E* is the event that the USG test result is malignant; *E*_1_ is the event that the same case has the FNAC test result positive; *E*′_1_ is the partition of the space for all cases from healthy (without any malignancy in the palpable thyroid mass) and it is a recorded value.

Here several probability values are, *p*(*E*) = probability of USG test malignancies = 0.15 from Table 3; *p*(*E*_1_) = probability of FNAC test malignancy (TP + FN) = 0.175 from Table 3. Thus, *p*(*E*|*E*_1_) = 0.15/0.175 = 0.857; *p*(*E*′_1_) = probability of TP = 0.125; *p*(*E*|*E*′_1_) = probability of (TP + TN) = 0.125 + 0.8 = 0.925.

As we seek the a posteriori probability value, substituting above values in its formula, *p*(*E*_1_|*E*) = *p*(*E*_1_) × *p*(*E*|*E*_1_)/[*p*(*E*_1_) × *p*(*E*|*E*_1_) + *p*(*E*′_1_) × *p*(*E*|*E*′_1_)], we get, *p*(*E*_1_|*E*) = (0.175 × 0.857)/[0.175 × 0.857) + (0.125 × 0.925)] = 0.14997/[0.14997 + 0.11562] = 0.564667.

Thus, *p*(*E*_1_|*E*) or *a posteriori probability* value = 0.564667 or 0.5647.

## Discussion

For a better stratification of malignant risk of TNs, this study deals with several suspicious USG features such as, hypoechoic texture, solid consistency, fine microcalcifications, irregular microlobulated margin, intranodular vascularity and absence of surrounding halo, as reported earlier.^4^, ^5^ Coexistence of at least two suspicious USG features is known to increase the risk of malignancy and further increase of the number of suspicious USG features increases the cancer risk many fold.^6^ Moreover, associated cervical lymphadenopathy as seen in this study also contributed to the increase of malignant risk, corroborating the report of Sanchez.^2^ Eventually, unnecessary FNAC of a majority of TNs are precluded.

Hyperplastic colloid nodules comprised of a major fraction, 80% (64 out of 80) of the total cases, as in a report.^7^ The majority (92%) of malignant TNs had female predominance (female: male ratio: 3:1) with a peak of incidence between 40 and 60 years of age, corroborating the report of Clark et al.^8^ Of the total, only 16 benign colloid nodule cases had features of multi-centricity presenting with additional nodules corroborating well with a study reported by Marquese et al.^9^ Solid and predominantly solid (containing less than 50% cystic areas) lesions had high predilection for being malignant.^10^, ^11^, ^12^ Echotexture of normal thyroid tissue remains slightly hyperechoic than the cervical strap muscles present anterior to the thyroid gland. Hypoechoic thyroid nodules appear relatively hypoechoic in comparison to these anterior strap muscles, as reported.^13^ Marked hypoechoic pattern was observed in 11 of 12 malignant nodules, thus favoring the diagnosis of malignancy. Around half of the benign lesions demonstrated hyperechogenicity, a clear indicator of benignity, while the rest of benign nodules appeared isoechoic.^12^, ^14^ None of the malignant nodules was having any purely hyperechoic or anechoic echotexture. Calcification of thyroid nodules was presented as both fine (micro) and coarse (macro) patterns, in 40% the total 80 cases. The highest incidence of fine micro-calcification was found in 11 of 12 malignant nodule cases, while 20 out of 21 calcified benign nodules demonstrated coarse macro-calcifications. The present study corroborates the presence of pertinent sonological features, anechoic or completely cystic structure, hyperechogenicity, and macro-calcifications were specific in classifying a nodule as benign.^15^ Incidence of malignancy was significantly higher in the calcified nodules.^16^, ^17^ Hence, it could be stated that a low incidence of malignancy can be predicted in patients with non-calcified thyroid nodules.

Thin complete perinodular sonolucent halo, caused by either the capsule or the surrounding vessels were demonstrated in 56 (including all the hyperechoic and isoechoic nodules) of 68 benign nodules. Thick incomplete or absence of peripheral halo was noted in the majority (82%) of malignant cases, thought to be due to the compression of normal thyroid tissue by the rapid growth of tumors^14^; perinodular thyroid parenchymal invasion too causes the absence of peripheral halo. Cystic degeneration was present in 16 cases of benign thyroid nodules, usually due to the colloid degeneration of hyperplastic nodules. Three cases of malignant nodules demonstrated intranodular cystic changes. In this study 10 out of 12 malignant nodule cases demonstrated central color flow pattern on CDFI, suggestive of intra-nodular vascularity in predominantly solid malignant nodules, as reported.^18^ Enlargement of multiple lymph nodes in the lateral cervical nodal chain was present in 10 of 12 malignant nodule cases. Most of the lymph nodes were rounded and hypoechoic, as compared to the thyroid tissue. Cystic cervical lymphadenopathy was noted in two papillary carcinoma cases.

All values, except likelihood ratios of Table 4, are cited herein as percentages. The prevalence (a priori probability or the pre-test probability or the presence of malignancy in the population) value was 17.5%, as there were a total of 14 FNAC malignancy (TP + FN) cases. And from incidences of both false cases (FN and FP), it was discernible that each test alone was insufficient for the diagnosis of malignancy. Further, both positive predictivity value (0.8333% or 83.33%, the fallibility of 16.67%) and negative predictivity value (0.9412% or 94.12%, a moderate fallibility of 5.88%) are computed, which are dependent on the prevalence of the disease; both these values indicated distinguishing efficiency of the USG test, the former for the presence and the later for the absence of malignancy, correctly.^19^ The former is the conditional probability value that a patient had the disease, given that the USG test result was indicative of malignancy. Similarly, the latter is the conditional probability that a patient did not have the malady, given that the USG test result indicated negativity for malignancy.

Moreover, two important test statistics, sensitivity and specificity are conditional of either having or not having the malignancy in a nodule of a patient and both are not affected by the prevalence value. The sensitivity of the USG test was 0.7143% or 71.43%; consequently, this figure strongly underrates the USG test to be an insufficient method for the diagnosis, in the presence of a stable malady. A high, exquisite value of the specificity (0.9697% or 96.97%), however, suggests an absolute dependency on the USG test at the absence of malignancy, on the contrary. Herein, to resolve this ambivalence, i.e., to provide an unbiased estimation of accuracy from the USG test, one would use the known correction method, based on sensitivity and specificity together, in a cumulative concept. In a balance, this value would be 84.2% [71.43 + 96.97)/2 = 84.2%]; in other words, for 84.2% cases the USG test would be dependable, for the diagnosis of thyroid malignancy in comparison to the FNAC test. In addition, the diagnostic accuracy value examines, by how common the USG test is effective and the moderately high accuracy value of 0.9250% or 92.5% signifies a proportionately high reliance on the USG test, for the subsequent management if malignancy is diagnosed, when the FNAC test result was still unknown; this value indicates accuracy of the USG test, regardless of positive or negative result of FNAC. Furthermore, the positive likelihood ratio of 2.3573 is a measure of the diagnostic accuracy from another angle; its magnitude indicated the certainty of a positive diagnosis of the USG test. Similarly, the negative likelihood ratio (0.2946) indicated its certainty of a negative diagnosis. Indeed, there would not be any dilemma on the USG test account, since a clinician inveterately advises for a USG test in case of a palpable thyroid mass, as a part of the routine preemptive practice. Thus, the 64 TN cases (80%) are justified. Admittedly, it is the most frequently followed preliminary method of diagnosis, rather safe and low-cost method being quick and non-invasive with the diagnostic protocol. Moreover, the amount of 02 FP cases (2.5%) of the total USG test results could be due to some insinuating human error of aspirating cells from the perinodular tissue missing the target nodule resulting in inadequate sampling during the guided FNAC test procedure. The assertive statement now would be that the USG and FNAC test had a 56.47% chance of correctly distinguishing a thyroid mass for presence/absence of malignancy, as inferred from the a posteriori probability value of 0.5647. If the pre-test probability (prevalence) of a disease would be lower as here, 17.5%, then the binary predictivity values of a positive test would be also lower. In this study, both the predictive values positive predictivity 83.33% and negative predictivity 94.12% indicate two independent assumptions: the USG test would be correct for 83.33% cases and the FNAC test would be accurate for 94.12% cases, when malignancy is prevalent in a patient. In the present case both the predictivity values are higher (positive 83.33% and negative 94.12%). So, from the negative predictive value of 94% (high level), the sufficiency of FNAC test is indicated, with a confidence. Moreover, the bandwagon of values of associated test statistics generated in the Bayesian analysis clump around the data-set, facilitating a multiple evaluation of the ambivalence with generated digital values of both tests. The value of a posteriori probability, 0.5647 from the post-test arithmetic computation of the data estimates a holistic diagnostic efficiency of both tests, and it specifically analyses how much (numerically) good each test is at independently arriving at the truth – the coveted conclusions from these tests. Thus, this analysis could provide a methodological framework of quantitative assessment of two test results of diagnosis of malignancy in a thyroid mass, as concluded for another issue.^20^

The TI-RADS is basically a USG classification. Normal thyroids with no evidence of focal lesion are classified as TI-RADS category 1, and benign thyroid nodules are the category 2; the category 3 includes probably benign thyroid nodules with <5% risk of malignancy.^2^ Undetermined nodules with 5–10% risk of malignancy are classified under the category TI-RADS 4a; TNs classified as category 4b are suspicious nodules with 10–50% risk of malignancy; and highly suspicious nodules with 50–85% risk of malignancy are classified as category 4c. The category TI-RADS5 includes probably malignant nodules.^4^, ^5^ The category TI-RADS 6 includes biopsy proved malignant TNs. Thyroid nodules of categories TI-RADS 3, 4a, 4b, 4c and 5 should always undergo FNAC for histological confirmation, except if contraindicated or in the event of high risk. In fact, color Doppler or elastography and scintigraphy or PET/CT could be used in suspected cases (probably benign) of the category 3, while in category 2 benign cases, FNAC and surgery would not be performed. Moreover, the requirement of the aggressive approach of FNAC may become redundant; nevertheless this is the gold standard test for diagnosis of malignancy. This work corroborates the recent developments of TI-RADS, with the most popular statistical view point with digital values of dependency on the USG assessment.

### Limitations of the statistical analysis

Several limitations of this analysis are signposted: (1) when the sensitivity value is higher, it would be easier to detect positivity (malignancy) in a population by the USG test; (2) high values of sensitivity and specificity are not affected by prevalence of the disease (malignancy) in a population; (3) albeit the specificity value was as high as 99% herein, it does not prompt that the accuracy of the USG test is equally high. Thus, neither the patient nor the clinician can get an answer for the question, what is the probability of the disease inferred from the positivity of the USG test; (4) as in the present study, there was as a low level of the error 2.5% (2 FP cases) insinuated into the FNAC test result, there should not be any objection for using it as the gold standard (verification bias) test.

## Conclusion

The majority of thyroid nodules were benign. Malignant thyroid nodules demonstrated USG characteristics of hypoechoic texture, fine micro-calcifications, solid consistency, internal vascularity and absence of surrounding halo. High sensitivity and specificity of the USG modality in relation to the gold standard guided FNAC results showed increasing importance of its diagnostic utility to differentiate malignant from benign lesions. Seen through Bayesian spectacles, it could be concluded that the USG test of a thyroid mass was efficient by 71.43–96.97%, in arriving at a positive result for the diagnosis of malignant thyroid nodules, when its FNAC test was positive. Herein, the prevalence of thyroid malignancy was 17.5%, while the post-test probability value with both USG and FNAC tests in differentiating malignant from the benign lesions was 56.47%, as inferred from the a posteriori probability value. Obviously, an early, accurate and hone diagnosis of thyroid malignancy still remains the mainstay of inclusion criteria for FNAB procedures and an important objective for a timely implementation of corrective surgical intervention, for the reduction of associated comorbidities as well as, the metastatic spread of this grisly disease to innards.

## Conflicts of interest

The authors declare no conflicts of interest.
